# Transcranial Magnetic Stimulation Over the Right Posterior Superior Temporal Sulcus Promotes the Feature Discrimination Processing

**DOI:** 10.3389/fnhum.2021.663789

**Published:** 2021-06-18

**Authors:** Qihui Zhou, Penghui Song, Xueming Wang, Hua Lin, Yuping Wang

**Affiliations:** ^1^Department of Neurology, Xuanwu Hospital, Capital Medical University, Beijing, China; ^2^Collaborative Innovation Center for Brain Disorders, Institute of Sleep and Consciousness Disorders, Beijing Institute of Brain Disorders, Capital Medical University, Beijing, China; ^3^Beijing Key Laboratory of Neuromodulation, Beijing Municipal Science and Technology Commission, Beijing, China

**Keywords:** transcranial magnetic stimulation, posterior superior temporal sulcus, dual-feature matching task, event-related potentials, electroencephalography

## Abstract

Attention is the dynamic process of allocating limited resources to the information that is most relevant to our goals. Accumulating studies have demonstrated the crucial role of frontal and parietal areas in attention. However, the effect of posterior superior temporal sulcus (pSTS) in attention is still unclear. To address this question, in this study, we measured transcranial magnetic stimulation (TMS)-induced event-related potentials (ERPs) to determine the extent of involvement of the right pSTS in attentional processing. We hypothesized that TMS would enhance the activation of the right pSTS during feature discrimination processing. We recruited 21 healthy subjects who performed the dual-feature delayed matching task while undergoing single-pulse sham or real TMS to the right pSTS 300 ms before the second stimulus onset. The results showed that the response time was reduced by real TMS of the pSTS as compared to sham stimulation. N270 amplitude was reduced during conflict processing, and the time-varying network analysis revealed increased connectivity between the frontal lobe and temporo-parietal and occipital regions. Thus, single-pulse TMS of the right pSTS enhances feature discrimination processing and task performance by reducing N270 amplitude and increasing connections between the frontal pole and temporo-parietal and occipital regions. These findings provide evidence that the right pSTS facilitates feature discrimination by accelerating the formation of a dynamic network.

## Introduction

Attention is the key cognitive function that selects currently relevant pieces of information at the expense of irrelevant ones, thereby facilitating the selection of features (Moore and Zirnsak, 2017). Classical studies of attention have identified that different attentional processing methods are carried out by two distinct attention systems: a dorsal system comprising the intraparietal sulcus and frontal eye fields, which are involved in top-down attention control, and a ventral feature-based system comprising the temporo-parietal junction for unexpected stimuli (Katsuki and Constantinidis, 2014; Majerus et al., 2018).

It is widely accepted that frontal and parietal cortices are the main brain areas related to attention control (Moore and Zirnsak, 2017), but recent evidence suggests that temporal cortex is also involved in the modulation of motion and color discrimination (Bogadhi et al., 2018; Stemmann and Freiwald, 2019) and is a critical structure in the cortical control of covert selective attention (Bogadhi et al., 2019). The posterior superior temporal sulcus (pSTS), as an essential part of temporal cortex, is thought to be involved in multiple neural processes (Hein and Knight, 2008; Beauchamp, 2011). Thus, clarifying the role of the pSTS in feature discrimination can provide a novel insight into the attention control system.

Transcranial magnetic stimulation (TMS) is a non-invasive brain stimulation method used to alter brain activity (Barker and Jalinous, 1985; Hallett, 2007). The TMS-induced spread of synchronized neural activity in the target area to connected brain regions can be observed by functional brain recording techniques (Siebner et al., 2009). Electroencephalography (EEG) has been increasingly employed in recent years to measure the cortical responses evoked by focal TMS at high temporal resolution (Bergmann et al., 2016). Combining TMS with EEG (TMS–EEG) enables the measurement of brain-wide cortical responses to TMS (Ilmoniemi and Kicic, 2010) and has permitted studies to examine the brain states and the dynamics across the cortical areas with excellent temporal resolution (Pellicciari et al., 2017). In addition, TMS–EEG provides precise information on the spatiotemporal order of activation of cortical areas, which can reveal causal interactions within functional brain networks (Rogasch and Fitzgerald, 2013).

Event-related potentials (ERPs) reflect brain functioning at a high temporal resolution (Luck, 2014). P100 (P1) and N150 (N1) are associated with early visual perceptual processing (Taylor, 2002; Luck, 2014), while P300 (P3) is related to information recognition and inhibition processing (Polich, 1998). N270 is a negative ERP component with a peak latency of around 270 ms that is elicited by conflicting modalities [e.g., visual and auditory (Wang et al., 2002), color and shape (Wang et al., 2004) and position mismatch (Yang and Wang, 2002)]. N270 amplitude is modulated by attention (Mao and Wang, 2008; Zhang et al., 2013). Thus, N270 is generally regarded as an electrophysiological marker of attention (Wang et al., 2001; Scannella et al., 2016). The dual-feature delayed matching task is associated with conflict processing (Wang et al., 2003; Zhang et al., 2003). Previous studies have revealed that conflict processing is modulated by attention (Carter et al., 2000), so we could use the task to explore the attentional modulation of pSTS to the conflict processing.

In this study, we used a single-pulse TMS–EEG procedure combined with a dual-feature delayed matching task to explore the role of the right pSTS in feature discrimination. We hypothesized that TMS applied over the right pSTS would modulate the brain dynamic network and facilitate the processing of the color-shape feature discrimination.

## Materials and Methods

### Participants

We recruited 21 healthy right-handed subjects [11 females; mean age (±SD): 24.2 ± 1.9 years] for the study. All subjects had a normal or corrected-to-normal vision with no color blindness and no history of any major diseases or neurological or mental disorders. Written informed consent was obtained from all participants, and the experiment was approved by the Ethics Committee of the Xuanwu Hospital, Capital Medical University.

### Experimental Procedures

All of the participants underwent a MRI scan and TMS. T1-weighted anatomic MRI was performed using a 3T MRI scanner (Siemens Medical Solutions, Erlangen, Germany) before the TMS to identify the target site for stimulation. Each participant completed two TMS sessions on 2 different days (for sham and real TMS sessions). Each TMS session comprised of four blocks, namely, two color and two shape task blocks; and each block consisted of 80 trials and lasted approximately 8 min.

During the two TMS sessions, participants sat on a comfortable chair in a dimly lit and quiet room with their gaze fixated on the computer monitor, completing both color and shape tasks in each TMS session. The order of tasks and TMS sessions was counterbalanced across subjects.

The resting motor threshold (RMT) of each participant was measured before the start of each session with an EEG cap. The RMT was defined as the lowest stimulus intensity that induced at least five motor evoked potentials of 50 μV over ten consecutive pulses in the first dorsal interosseous muscle.

### Experimental Task

**Figure 1:**
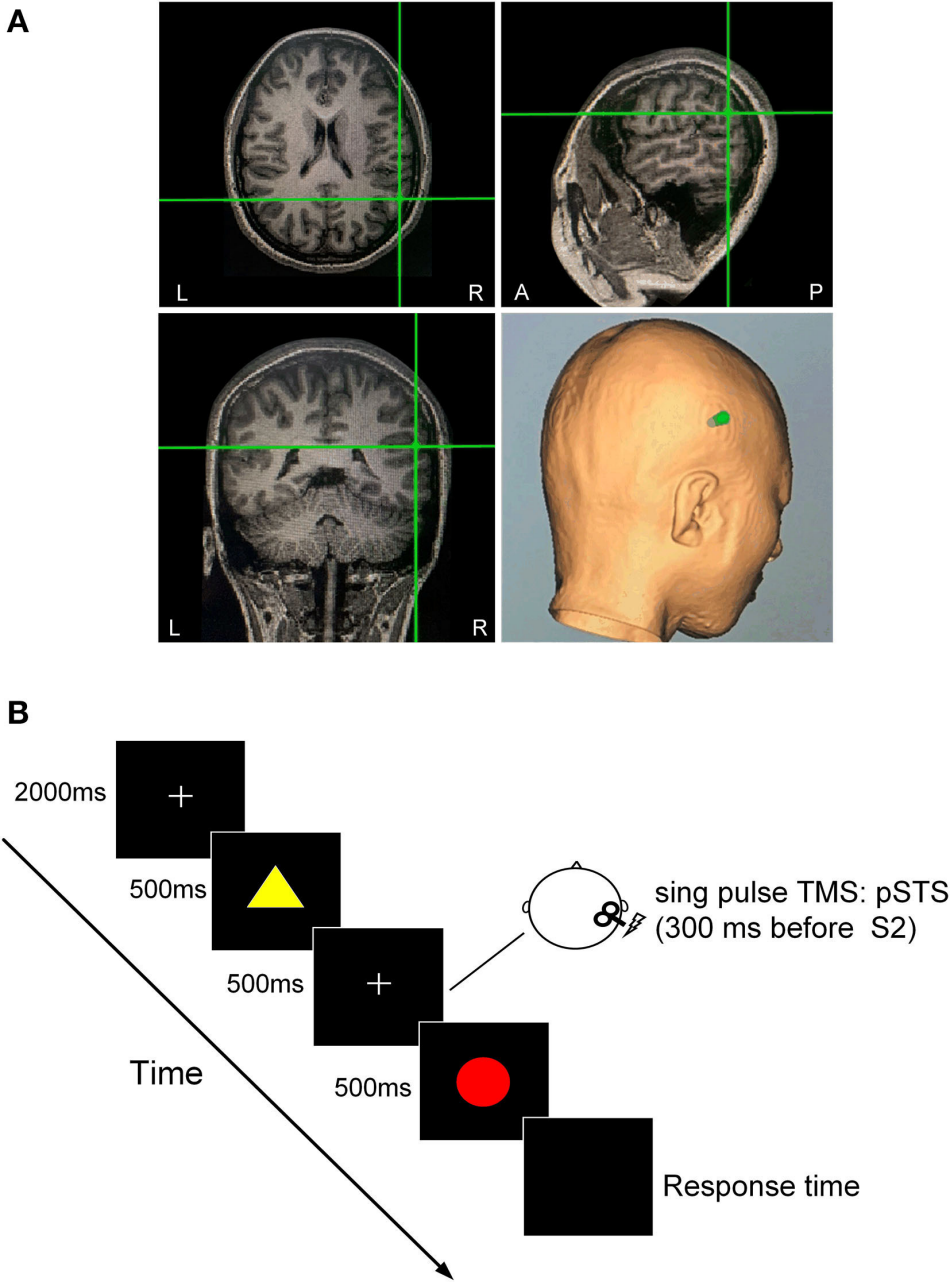
Dual-feature delayed matching task. **(A)** TMS-targeted areas of the right pSTS in one subject. The TMS coil orientation was determined based on the anatomical MRI scan. **(B)** For each trial, two presentations of the first (S1) and second (S2) stimuli were separated by 500 ms, during which a sham or real TMS pulse was applied over the right pSTS 300 ms before the onset of S2 presentation. At the end of the trial, the subject pressed the left or the right button of a mouse to judge whether the color of S2 was identical to that of S1while ignoring the shape of the stimulus in the color task, or whether the shape of S2 was identical to that of S1 while ignoring the color of the stimulus in the shape task.

Each participant performed a dual-feature delayed matching task (Wang et al., 2004; Figure 1). A stimulus presentation system (STIM; Neurosoft Labs, Charlotte, NC, the United States) was used to present each target in the task, which was defined by the combination of a specific color (red, green, yellow, or white) and shape (pentagon, ellipse, triangle, hexagon, octagon, arrow, cross, circular, pentagram, or rhombus).

Each trial began with a central fixation point presented for 2,000 ms. The first (S1) and second (S2) stimuli were presented for 500 ms each with an interstimulus interval of 500 ms. A single sham or real TMS pulse was delivered 300 ms before the onset of S2 in each trial. The interval between the end of the previous S2 and the onset of the next S1 was 5 s. Stimuli were presented in the center of the screen on a black background. There were four types of stimulus pair: C–S– [same color, same shape (match)]; C + S– [different color, same shape (task-relevant mismatch in color task)]; C–S + [same color, different shape (task-irrelevant mismatch in color task)]; and C + S+ [different color, different shape (conjunction mismatch)]. The four types of stimulus pairs were randomly presented in sequence, and each type had the same probability.

The color task (selective attention to color, Ac) required participants to judge whether the color of S2 was identical to that of S1 while ignoring their shape. The shape task (selective attention to shape, As) required participants to discriminate whether S1 and S2 had identical shapes while ignoring their color. Upon the presentation of S2, participants were required to press the left or right button of a mouse with their left or right thumb to indicate their response as quickly and accurately as possible. The response hand was counterbalanced in each subject.

### TMS Site Localization

The TMS target sites in the right pSTS were individually identified using T1-weighted anatomic MRI image of the participants with Brainsight frameless stereotactic neuronavigation system (SofTaxic Navigator System, EMS Italy). For each participant, the right pSTS area was localized using anatomical features: the inflection point in the pSTS where it angles upward toward the parietal lobe (Beauchamp et al., 2008). We manually selected the inflection as the location of pSTS on the structural MRI image of an individual. A near-IR navigation system was used to track the position of the stimulation coil to identify the right pSTS in the head of the participant.

### TMS–EEG

Single-pulse TMS was delivered to the right pSTS using a monophasic Magstim stimulator (Magstim Company Ltd., London, the United Kingdom) with a figure-of-eight coil (outer winding diameter of 70 mm). The stimulation intensity was applied at 90% of the RMT. In the sham TMS condition, the intensity was the same but the surface of the coil was perpendicular to the head of the subject.

Continuous EEG recording from 21 scalp electrodes in an elastic cap that were positioned according to the international 10–20 system (Greentek Ltd, Wuhan, China) was performed with a magnetic field-compatible EEG amplifier (Yunshen, Beijing, China). The apex nasi was used as a reference. Skin impedance was maintained below 5 kΩ. The signal was digitized at a sampling rate of 1,024 Hz. Earplugs with white noise were used at all times by the participants to attenuate the sound of TMS.

### Data Analysis

#### Behavioral Data Analysis

Behavioral data were extracted from the perceptual reports. Trials with incorrect responses or a reaction time (RT) <200 or >1,500 ms were excluded from the analysis. The results from the two tasks were averaged across the 21 participants. The correct rate and mean RT data were subjected to repeated-measures analysis of variance (ANOVA) with condition (real/sham), task (color/shape), and type (match/task-relevant/task-irrelevant/conjunction mismatch) as within-subject factors.

#### ERP Data Analysis

The EEG data were analyzed with MATLAB vR2015b (MathWorks, Natick, MA, the United States) using customized scripts and EEGLAB Toolbox (Delorme and Makeig, 2004). The preprocessing consisted of two rounds of independent component analysis (ICA) (Hyvärinen and Oja, 2000): the first to remove the components containing large-amplitude TMS-induced artifacts and the second to remove any remaining artifacts (e.g., blinking). TMS-induced artifacts were removed by discarding the signal for 60 ms after each pulse. The EEG epochs were extracted using a time window of 2,000 ms (1,000 ms before and 1,000 ms after TMS) and baseline-corrected to the 200 ms before the first visual stimulus onset.

The amplitude of P1, N1, N270, and P3 was measured from the averaged waveforms at notable electrodes. By the visual inspection of the ERPs, the mean amplitudes of P1 (60–110 ms), N1 (112–200 ms), N270 (220–320 ms), and P3 (322–500 ms) in corresponding notable electrodes were analyzed. Electrophysiological parameters were analyzed by means of a four-way ANOVA with conditions and task, type, and electrode sites as factors. The Greenhouse–Geisser epsilon correction for non-sphericity was applied where appropriate. *Post-hoc* paired *t*-tests were passed through Bonferroni correction for multiple comparisons.

#### Time-Varying Network Analysis

EEG data analysis was divided into preprocessing and time-varying network analysis. The latter required several segmentations to enable the construction of a reliable network that captured the brain architectures and networks. In this study, we used TMS disturbances as stimulus labels. For each labeled disturbance event, the time point corresponding to the peak of the label was set as time 0; data corresponding to 0.5 s before and 1 s after time 0 were extracted (total segment length = 1.5 s).

To reduce the calculation load in the time-varying network analysis, eight times downsampling was applied to obtain a sampling frequency of 32 Hz. The time-varying network was calculated by the adaptive directed transfer function (ADTF) method and a time-varying multivariate adaptive autoregressive (tv-MVAAR) model (Zhang et al., 2017) to observe dynamic information processing during TMS disturbance. The two-sample *t*-test was used to compare time-varying networks under the sham and real TMS conditions, and false discovery rate correction was applied for multiple comparisons. *P* < 0.05 were regarded as statistically significant.

#### Time-Varying Multivariate Adaptive Autoregressive Model

For each artifact-free segment, the tv-MVAAR model is defined as

(1) $$\begin{array}{r} X\left(t\right)=\sum_{i=1}^{p}A\left(i,t\right)X\left(t-1\right)+E\left(t\right) \end{array}$$

where *X(t)* represents the data vector of EEG signal and *E(t)* represents the multivariate independent white noise. *A(i,t)* represents the matrix of tv-MVAAR model coefficients, which is estimated by the Kalman filter algorithm. *p* represents the order of the model that is automatically determined by the Akaike Information Criterion (AIC) within the range of 2–20 as,

(2) $$\begin{array}{r} AIC\left(p\right)=In\left[\det\left(\chi \right)\right]+{2M}^{2}p/N \end{array}$$

where *M* is the number of the electrodes, *p* is the optimal order of the model, *N* represents the number of the time points of each time series, and χ represents the corresponding covariance matrix.

#### Adaptive Directed Transfer Function

Parameters *A(f,t)* and *H(f,t)* in the frequency domain are defined as follows:

(3) $$\begin{array}{rcl} A\left(f,t\right) & = & \sum_{k=0}^{p}A_{k}\left(t\right)e^{-j2\pi ftk} \end{array}$$

(4) $$\begin{array}{rcl} A\left(f,t\right)X\left(f,t\right) & = & E\left(f,t\right) \end{array}$$

(5) $$\begin{array}{rcl} X\left(f,t\right) & = & A^{-1}\left(f,t\right)E\left(f,t\right)=H\left(f,t\right)E\left(f,t\right) \end{array}$$

where *A*_*k*_ is the matrix of the tv-MVAAR model coefficients, and *X(f,t)* and *E(f,t)* are the Fourier transformations of *X(t)* and *E(t)* in the frequency domain, respectively.

Moreover, the normalized ADTF describing the directed flow from the *j*th to the *i*th node is defined in Equation (6), and the final integrated ADTF is defined in Equation (7) within the frequency band of interest (i.e., 0.5–14.5 Hz in this work) as follows:

(6) $$\begin{array}{rcl} \gamma_{ij}^{2}\left(f,t\right) & = & \frac{|H_{ij}\left(f,t\right){|}^{2}}{\sum_{m=1}^{n}|H_{im}\left(f,t\right){|}^{2}} \end{array}$$

(7) $$\begin{array}{rcl} Q_{ij}^{2}\left(t\right) & = & \frac{\sum_{k=f_{1}}^{f_{2}}\gamma_{ij}^{2}\left(k,t\right)}{f_{2}-f_{1}} \end{array}$$

The normalized total information outflow of the *j*th node is further estimated in Equation (8) as:

(8) $$\begin{array}{r} Q_{j}^{2}\left(t\right)=\frac{\sum_{k=1}^{n}Q_{kj}^{2}\left(t\right)}{n-1}\;,\;for\;k\neq j \end{array}$$

where *n* is the total number of nodes. When each node (*n*) has been calculated for each sample time point (*t*), a directional edge (*i* to *j*) can be displayed. From Equation 8, we can derive an outflow that denotes the time-varying of each node across different time points.

## Results

### Behavior Data

The correct rate and mean RTs in the dual-feature delayed matching task are shown in Table 1. The 2 × 2 × 4 ANOVA of RTs revealed the main effects of condition [*F*_(1, 20)_ = 26.87, *P* < 0.001] and stimulus type [*F*_(3, 60)_ = 15.47, *P* < 0.001]. A *post-hoc* analysis showed that RT was significantly shorter in the real TMS condition than in the sham TMS condition with all types (*Ps* < 0.05). In both conditions, RTs were significantly longer for task-relevant and conjunction mismatch types than for the match type in each task (*P* < 0.05). The overall correct response rates for each type and task did not differ between the two conditions (*P* > 0.05).

**Table 1:** Mean reaction time and correct rate in two conditions for each task and type.

	**Type^†^**	**Sham TMS**		**Real TMS**	
		**Ac**	**As**	**Ac**	**As**
Reaction time (ms)	Match	573.8 ± 72.8	555.6 ± 90.2	527.4 ± 81.8^*^	524.4 ± 85.5^*^
	Task-relevant	616.4 ± 66.7^a^	638.9 ± 64.2^a^	572.9 ± 84.6^*^^a^	602.8 ± 77.7^*^^a^
	Task-irrelevant	559.3 ± 71.1^b^	574.5 ± 81.0^b^	533.8 ± 87.5^*^	547.4 ± 77.6^*^^b^
	Conjunction	623.6 ± 62.5^a^^c^	614.7 ± 75.7^a^^c^	578.2 ± 88.9^*^^a^	599.3 ± 85.8^a^^c^
Correct rate (%)	Match	98.3 ± 2.1	99.3 ± 1.8	99.0 ± 1.7	99.2 ± 1.4
	Task-relevant	99.2 ± 1.4	98.6 ± 2.0	99.0 ± 1.8	98.6 ± 2.0
	Task-irrelevant	99.0 ± 1.5	99.1 ± 1.6	99.1 ± 1.6	98.1 ± 2.0
	Conjunction	98.4 ± 2.0	99.2 ± 1.4	99.3 ± 1.1	98.3 ± 2.6

*Data represent mean ± SD*.

* *Significantly different from sham condition in the same task and type (P < 0.05)*.

a *Significantly different from match type in the same condition and task (P < 0.05)*.

b *Significantly different from the relevant type in the same condition and task (P < 0.05)*.

c *Significantly different from the irrelevant type in the same condition and task (P < 0.05)*.

† *Match, Ac/C–S– or As/C–S–; task-relevant, Ac/C + S– or As/C–S +; task-irrelevant, Ac/C–S+ or As/C+S–; conjunction, Ac/C + S + or As/C + S+; C–S–, color and shape are the same in stimulus pair; C–S+, the same color with different shape; C + S–, different color with the same shape; C+S+, color and shape both are different*.

*Ac, attention to color; As, attention to shape; TMS, transcranial magnetic stimulation*.

### ERP data

#### Grand-Averaged ERP Waveforms

**Figure 2:**
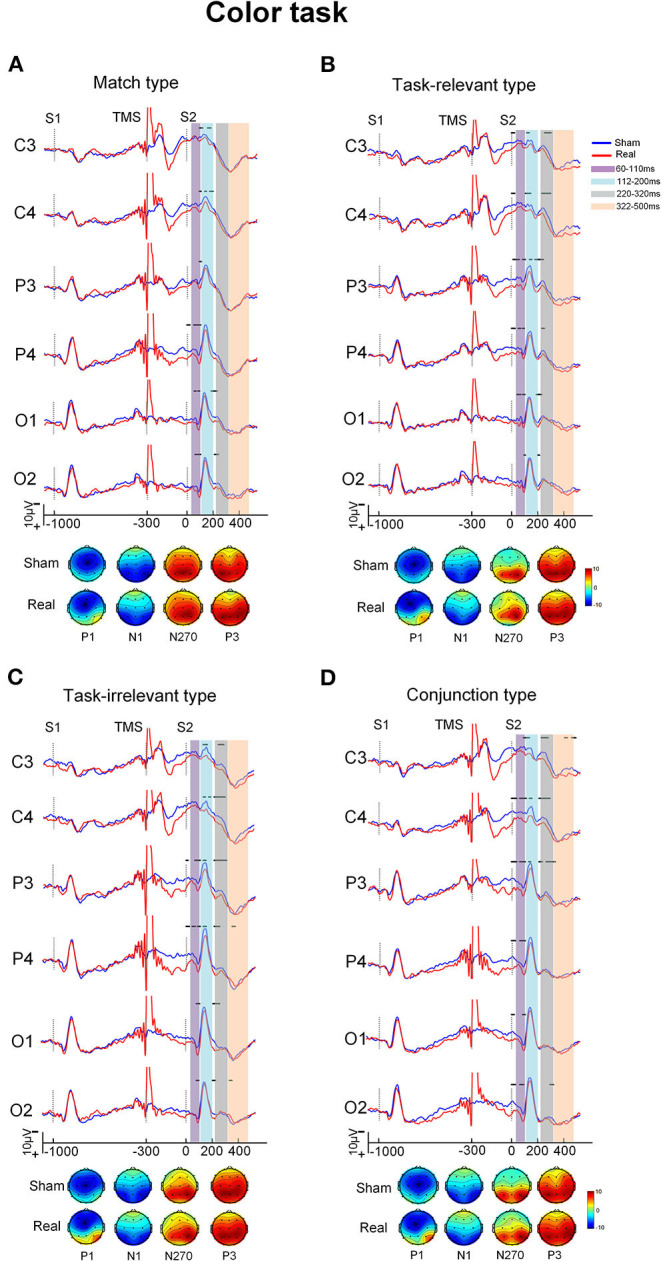
The grand-averaged ERPs and topographies of each type and condition in color task. The ERPs showed difference between the sham and real TMS condition [upper of **(A–D)**]. The amplitude of P1 in real TMS condition was increased than that in sham TMS condition, while the amplitude of N270 was reduced in mismatch types. The topographies [bottom of **(A–D)**] showed that P1 at posterior scalp areas in real TMS condition was more positive than sham one. Vertical lines indicated the TMS, S1, and S2 stimulus onset. Horizontal solid line indicates the time after the onset of S2 with statistical significance between the two conditions. The purple-shaded areas indicate the time window (60–110 ms) in which the P100 was measured, the blue-shaded areas indicate the time window (112–200 ms) in which the N150 was measured, the gray-shaded areas indicate the time window (220–320 ms) in which the N270 was measured, and the orange-shaded areas indicate the time window (322–500 ms) in which the P3000 was measured.

**Figure 3:**
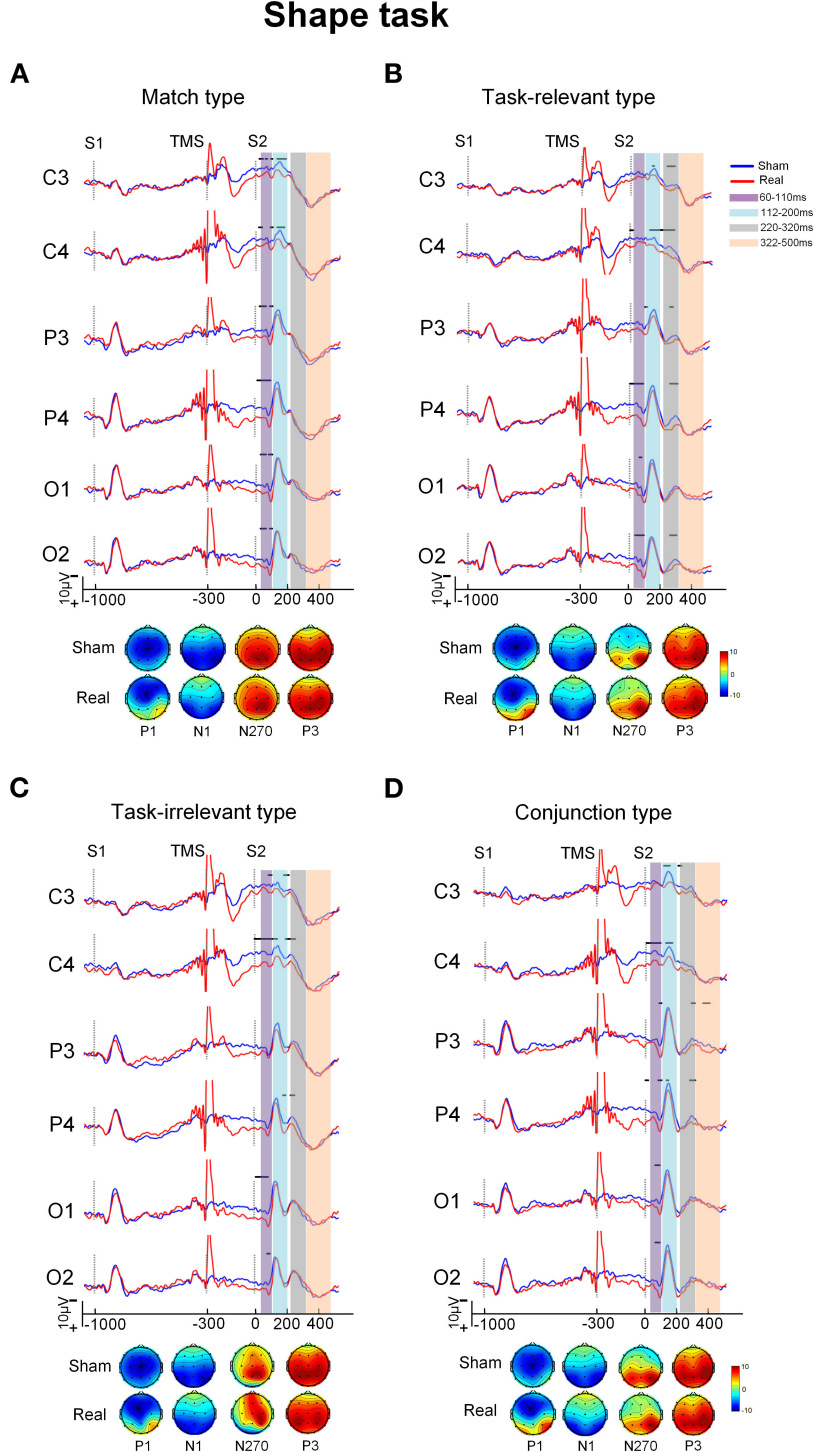
The grand-averaged ERPs and topographies of each type and condition in shape task. The ERPs showed difference between the sham and real TMS condition [upper of **(A–D)**]. The amplitude of P1 in real TMS condition was increased than that in sham TMS condition, while the amplitude of N270 was reduced in mismatch types. The topographies [bottom of **(A–D)**] showed that P1 at posterior scalp areas in real TMS condition was more positive than in sham one. Vertical lines indicated the TMS, S1, and S2 stimulus onset. Horizontal solid line indicates the time after the onset of S2 with statistical significance between the two conditions. The purple-shaded areas indicate the time window (60–110 ms) in which the P100 was measured, the blue-shaded areas indicate the time window (112–200 ms) in which the N150 was measured, the gray-shaded areas indicate the time window (220–320 ms) in which the N270 was measured, and the orange-shaded areas indicate the time window (322–500 ms) in which the P3000 was measured.

Grand-averaged ERP waveforms difference between the real and sham conditions for the four types in each task are shown in Figures 2, 3. The ERPs with the four types consisted of P1 and N1 components at the posterior scalp and the P3 component over the whole scalp. The amplitude of P1 in the real TMS condition was increased than that in the sham TMS condition, while the amplitude of N270 was reduced in mismatch types. Visual inspection of the grand-averaged data showed mean amplitudes measured at 60–110 ms for P1, 112–200 ms for N1, 220–320 ms for N270, and 322–500 ms for P3. The components were similar in the sham and real TMS conditions.

#### P1 Component

In the time window between 60 and 110 ms, after the onset of S2, the condition showed a main effect on the amplitude [*F*_(1, 20)_ = 10.32, *P* = 0.004] as well as interaction with electrode area [*F*_(3, 60)_ = 10.08, *P* < 0.001). The pairwise comparisons indicated that the amplitude at electrode P4 was more positive in the real TMS condition than in the sham condition (*P* < 0.05; Table 2).

**Table 2:** Mean amplitude with all stimulus types for each task and condition in different time windows.

**Time window**	**Condition**	**Area**	**Ac**				**As**			
			**Match**	**Task-relevant**	**Task-irrelevant**	**Conjunction**	**Match**	**Task-relevant**	**Task-irrelevant**	**Conjunction**
60–110 ms	Sham TMS	P3	−3.5 ± 7.7	−4.2 ± 6.8	−3.0 ± 7.4	−2.5 ± 5.5	−4.2 ± 6.0	−3.6 ± 8.4	−5.2 ± 5.5	−3.2 ± 8.7
		P4	−3.4 ± 8.0	−3.3 ± 7.1	−2.3 ± 8.2	−2.3 ± 6.6	−4.3 ± 6.8	−2.5 ± 5.5	−5.1 ± 7.0	−3.4 ± 7.6
		O1	−0.6 ± 8.5	−1.2 ± 7.0	−0.9 ± 9.3	−0.3 ± 7.6	−2.0 ± 8.3	−0.4 ± 6.2	−2.5 ± 8.2	−1.5 ± 9.8
		O2	−1.3 ± 9.1	−0.9 ± 6.9	−1.1 ± 9.4	−1.0 ± 8.1	−5.2 ± 7.0	−0.8 ± 7.0	−3.5 ± 9.1	−1.8 ± 9.6
	Real TMS	P3	−2.8 ± 6.5	−0.6 ± 7.7^*^	−1.4 ± 6.5	−1.1 ± 6.7	−2.0 ± 4.4	−0.9 ± 5.7	−2.9 ± 5.1	−0.1 ± 4.2^*^
		P4	−0.1 ± 7.5^*^	1.9 ± 8.0^*^	2.1 ± 8.0^*^	2.3 ± 6.5^*^	1.2 ± 6.3^*^	3.0 ± 6.6^*^	1.9 ± 6.7^*^	2.8 ± 5.6^*^
		O1	0.2 ± 8.2	1.2 ± 7.9	2.8 ± 9.0^*^	1.0 ± 8.8	0.3 ± 7.1	3.5 ± 7.3^*^	0.1 ± 7.7	1.0 ± 8.3
		O2	0.7 ± 8.3	0.8 ± 6.6	1.0 ± 8.1	0.9 ± 8.1	0.4 ± 8.4	3.9 ± 7.9^*^	1.9 ± 5.7^*^	1.4 ± 7.4
112–200 ms	Sham TMS	P3	−7.4 ± 6.4	−6.2 ± 5.1	−7.6 ± 6.6	−5.4 ± 7.2	−8.1 ± 5.8	−7.0 ± 7.8	−6.9 ± 5.1	−6.4 ± 7.4
		P4	−8.5 ± 6.4	−6.4 ± 7.8	−8.8 ± 6.8	−5.4 ± 6.5^a^^c^	−9.2 ± 6.8	−7.8 ± 7.0	−8.0 ± 6.5	−5.1 ± 8.8^a^^b^^c^
		O1	−10.7 ± 7.8	−9.5 ± 7.1	−10.9 ± 7.8	−8.1 ± 6.3^a^^c^	−11.2 ± 7.2	−9.7 ± 6.9	−9.8 ± 6.7	−10.6 ± 8.4
		O2	−10.4 ± 8.7	−9.1 ± 9.8	−11.2 ± 9.1	−9.1 ± 9.5	−10.6 ± 5.1	−10.6 ± 7.7	−9.5 ± 9.3	−10.6 ± 9.9
	Real TMS	P3	−6.8 ± 5.7	−5.1 ± 5.3	−5.6 ± 5.9	−4.4 ± 5.2^a^	−6.0 ± 5.0	−5.1 ± 4.7	−4.8 ± 6.5	−6.5 ± 5.9
		P4	−6.8 ± 8.0	−5.6 ± 5.3	−6.2 ± 8.4	−3.2 ± 6.8^a^^c^	−8.3 ± 8.7	−4.7 ± 6.0	−8.4 ± 4.5^b^	−5.1 ± 7.7^a^^c^
		O1	−10.1 ± 8.0	−8.5 ± 6.7	−9.9 ± 7.1	−8.6 ± 6.9	−9.4 ± 7.5	−8.6 ± 5.9	−8.1 ± 6.5	−8.6 ± 5.9
		O2	−8.9 ± 9.5	−7.2 ± 7.9	−9.3 ± 8.9	−8.3 ± 8.1	−7.7 ± 7.6	−8.8 ± 7.2	−8.0 ± 8.4	−8.8 ± 6.4
220–320 ms	Sham TMS	C3	6.1 ± 7.8	−1.6 ± 3.9^a^	1.0 ± 4.7^a^^b^	−1.0 ± 5.4^a^	6.2 ± 7.6	0.5 ± 5.0^a^	1.9 ± 5.1^a^	1.0 ± 5.5^a^
		C4	6.7 ± 7.8	0.1 ± 6.1^a^	2.0 ± 6.3^a^	0.5 ± 4.4^a^	6.2 ± 8.0	0.5 ± 5.6^a^	2.0 ± 5.4^a^	1.5 ± 4.1^a^
		P3	8.2 ± 9.7	0.4 ± 4.2^a^	4.7 ± 5.0^b^	3.3 ± 5.6^a^^b^	7.0 ± 9.4	2.9 ± 5.4	2.5 ± 6.8^a^	3.8 ± 4.2
		P4	8.1 ± 9.3	2.1 ± 5.0^a^	6.1 ± 5.3^b^	3.2 ± 5.8^c^	8.0 ± 9.8	3.9 ± 8.5^a^	2.8 ± 7.2^a^	4.9 ± 5.2
	Real TMS	C3	6.5 ± 6.2	1.5 ± 5.1^*^^a^	4.0 ± 5.8^*^^a^^b^	2.4 ± 5.6^*^^a^	6.0 ± 6.4	3.1 ± 4.8^*^^a^	3.8 ± 5.4^*^^a^	3.6 ± 5.1^*^^a^
		C4	8.5 ± 5.9	5.7 ± 5.7^*^^a^	5.4 ± 7.2^*^^a^	3.5 ± 5.2^*^^a^	7.5 ± 6.7	4.5 ± 5.8^*^^a^	5.0 ± 5.1^*^^a^	4.3 ± 5.2^*^^a^
		P3	8.7 ± 7.0	3.7 ± 5.3^*^^a^	7.1 ± 3.0^*^^b^	5.8 ± 5.2^*^^b^	6.6 ± 7.5	6.1 ± 5.3^a^	3.7 ± 6.9^*^	6.6 ± 5.0^*^
		P4	9.9 ± 7.1	5.2 ± 5.4^*^^a^	9.1 ± 4.8^*^^b^	6.0 ± 5.7^*^^a^^c^	8.4 ± 7.9	7.5 ± 7.0^*^^a^	5.6 ± 6.0^*^	7.4 ± 5.0^*^
322–500 ms	Sham TMS	C3	−7.4 ± 4.9	7.8 ± 3.4	8.6 ± 4.4	8.1 ± 3.5	9.1 ± 5.5	8.6 ± 5.6	9.9 ± 4.9	7.7 ± 4.7
		C4	9.7 ± 4.6	8.9 ± 4.1	9.2 ± 3.6	8.7 ± 4.8	9.7 ± 5.3	9.7 ± 5.9	9.9 ± 5.2	8.7 ± 5.1
		P3	9.7 ± 5.4	9.7 ± 4.2	10.4 ± 5.4	9.5 ± 3.9	9.9 ± 5.4	10.0 ± 7.0	10.2 ± 5.6	8.7 ± 6.1
		P4	8.4 ± 6.3	9.3 ± 5.9	10.0 ± 4.8	8.9 ± 5.3	9.5 ± 6.3	10.7 ± 8.0	9.8 ± 5.1	8.3 ± 6.7
	Real TMS	C3	7.1 ± 4.4	8.3 ± 6.1	9.6 ± 5.0	7.4 ± 5.0	8.5 ± 3.9	7.2 ± 4.1	10.3 ± 3.9	6.8 ± 4.8
		C4	9.0 ± 4.6	10.2 ± 6.1	10.2 ± 3.9	9.5 ± 5.7	9.8 ± 4.0	9.6 ± 5.3	10.9 ± 4.0	8.9 ± 5.5
		P3	9.7 ± 5.0	10.7 ± 5.7	10.6 ± 6.0	9.6 ± 5.2	9.6 ± 4.9	8.9 ± 4.6	12.0 ± 5.6	8.4 ± 5.3
		P4	9.4 ± 5.0	10.6 ± 5.2	9.9 ± 5.3	9.4 ± 5.5	9.3 ± 5.2	9.9 ± 6.0	11.2 ± 5.6	8.8 ± 6.1

*Data represent mean ± SD*.

* *Significantly different from sham condition in the same type and areas of each task (P < 0.05)*.

a *Significantly different from match type in the same condition and areas of each task (P < 0.05)*.

b *Significantly different from the relevant type in the same condition and areas of each task (P < 0.05)*.

c *Significantly different from the irrelevant type in the same condition and areas of each task (P < 0.05)*.

*Ac, attention to color; As, attention to shape; TMS, transcranial magnetic stimulation*.

#### N1 Component

In the time window between 112 and 200 ms after the onset of S2, the stimulus type showed main effects on the amplitude [*F*_(3, 60)_ = 4.63, *P* = 0.006]. Type also showed an interaction with electrode area [*F*_(9, 180)_ = 3.78, *P* < 0.001]. The *post-hoc* analysis showed that the conjunction type was more positive than match and irrelevant types at electrode P4 (*P* < 0.05). However, no significant difference of the amplitude between the real and sham TMS conditions was found [*F*_(1, 20)_ = 3.25, *P* = 0.087; Table 2).

#### N270 Component

In the time window between 220 and 320 ms, ANOVA revealed the significant main effects of condition [*F*_(1, 20)_ = 20.40, *P* < 0.001] and type [*F*_(1.35, 27.05)_ = 11.02, *P* = 0.001] on the amplitude. Type showed an interaction with electrode [*F*_(5.27, 105.30)_ = 2.92, *P* = 0.015]. The *post-hoc* analysis showed that the amplitude of the mismatch types was more negative than that of the match type at electrodes C3 and C4 (*P* < 0.05).

Condition also showed an interaction with type [*F*_(1.50, 29.96)_ = 4.85, *P* = 0.023]. The pairwise comparisons revealed that amplitude was significantly more positive in the real TMS condition than in the sham TMS condition with all three mismatch types in each task (all *Ps* < 0.05). However, in the match type, there was no significant difference in amplitude between the sham and real TMS conditions (Table 2).

#### P3 Component

In the time window between 322 and 500 ms, the mean amplitude of P300 did not differ between the sham and real TMS conditions [*F*_(1, 20)_ = 0.16, *P* = 0.693]. The TMS condition did not interact with task [*F*_(1, 20)_ = 0.24, *P* = 0.629], type [*F*_(3, 60)_ = 0.74, *P* = 0.533], or electrode [*F*_(1.66, 33.25)_ = 1.41, *P* = 0.257; Table 2].

#### Topography

Viewing the topography of the two conditions, it showed that the amplitude of P1 at different scalp areas in the real TMS condition was more positive than that in the sham TMS condition in both tasks. The N1, N270, and P3 distributions have showed no distinct differences between the sham and real TMS conditions in both tasks (Figures 2, 3).

### Time-Varying Network

**Figure 4:**
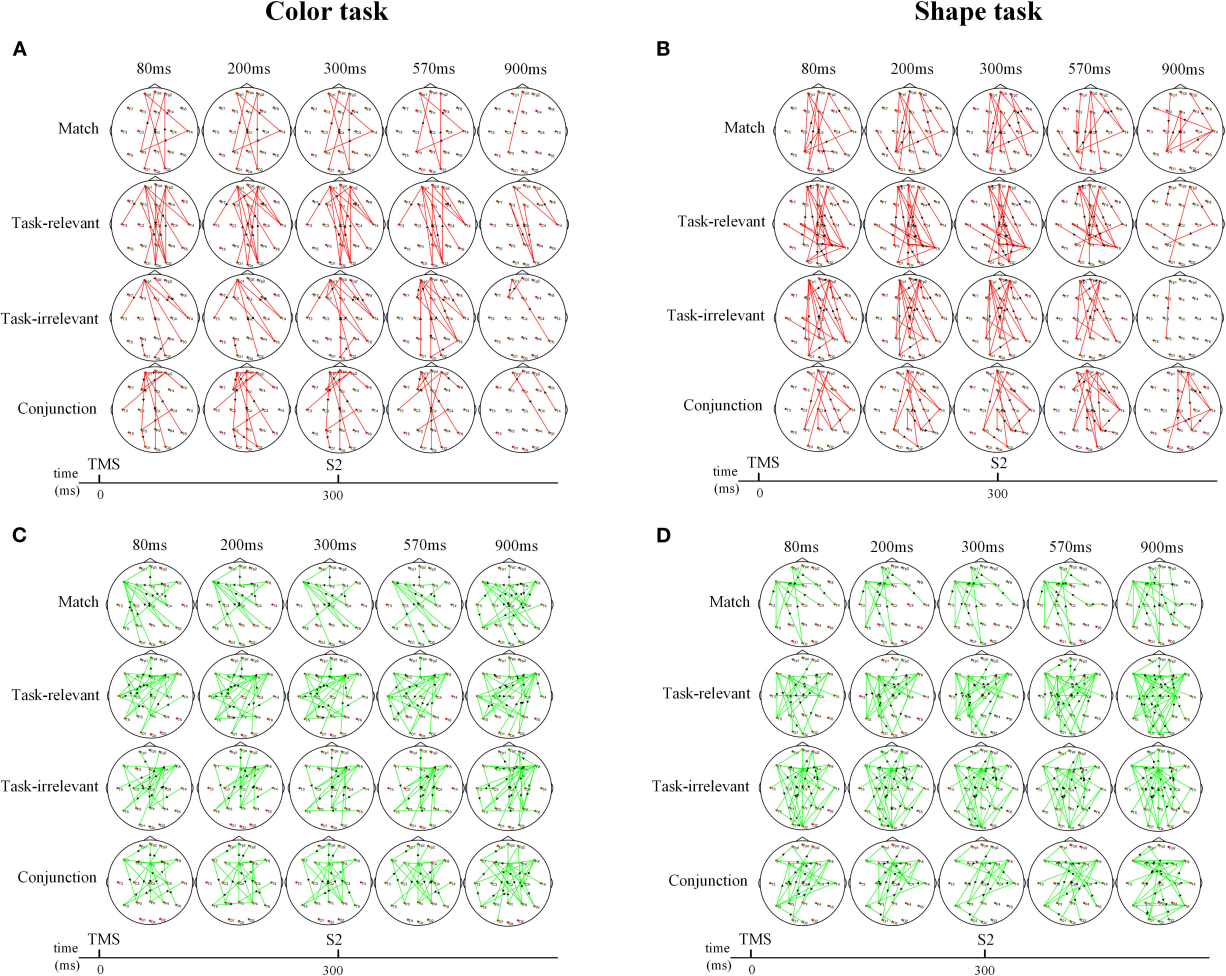
The time-varying network changes between sham and real TMS in color and shape task. **(A,B)** Increased network connections between sham and real TMS condition. **(A)** The connections between the bilateral frontal pole and temporo-occipital region were significantly increased in the color task**. (B)** The connections between the bilateral frontal pole and temporo-occipital, parietal region were significantly increased in the shape task. **(C,D)** Decreased network connections between sham and real TMS condition. **(C)** The connections between the bilateral frontal region and temporo-parietal region, central region were significantly reduced in the color task. **(D)** The information outflows in the frontal and mid-line region were significantly reduced. Red and green lines indicate the increased connection and decreased connection, respectively. Arrows indicate the direction of information flow.

Changes in the time-varying network differed significantly between sham and real TMS conditions (Figure 4). TMS of the right pSTS induced changes in the time-varying networks of different types. The increased and decreased connections by real TMS appeared at the timing of TMS and are maintained throughout S2 presentation. Here, we focused on the conflict processing stage, which was around 270 ms after the presentation of S2.

In the color task, the connections between the bilateral frontal poles and the temporo-occipital region were significantly increased with each type compared to the sham TMS condition (Figure 4A). Meanwhile, the connections between the bilateral frontal regions and the temporo-parietal region, central regions, were decreased in each type (Figure 4B).

In the shape task, the connections between the bilateral frontal poles and the temporo-occipital region, parietal regions, were increased in each type (Figure 4C). Meanwhile, compared to the sham TMS condition, the information outflows in the frontal and midline region were significantly reduced in the real TMS condition (Figure 4D).

## Discussion

The results of this study demonstrate that, in addition to the frontal and parietal cortices, the pSTS makes a significant contribution to attentional processing in humans. Furthermore, we found that single-pulse TMS of the pSTS facilitates feature discrimination, possibly by promoting the formation of a dynamic attention network.

### Behavior Performance

In this experiment, the better performance in RTs was not at the cost of correct response in the real TMS condition. Previous studies have reported that behavioral changes were facilitated or inhibited by TMS, depending on the stimulated region or parameters (Shapiro et al., 2001; Cappa et al., 2002).

The results of this study demonstrate that single-pulse TMS of the pSTS facilitated task performance, as the temporal areas have been claimed to be associated with perceptual discrimination (Lambert and Wootton, 2017). The positive outcome on behavior may result from the excitatory effects of TMS on neurons in the pSTS stimulated area (Pascual-Leone et al., 2000). However, Sack and Linden showed that TMS-induced changes in behavior may be ascribed not only to the stimulated area but also to the network of areas connected to the stimulated site (Sack and Linden, 2003). Thus, the greater performance may also probably result from the facilitation of intracortical networks associated with feature discrimination. The overall correct response rates for each type and task in two conditions were over 98%. It is noteworthy that although the RT showed the difference between sham and real TMS, the correct rate did not show a significant difference, which may be due to a ceiling effect.

### P1 and N1

P1 and N1 are the earliest components linked to visual perceptual processing. N1 is related to the orientation of attention to relevant stimuli, and the P1 component reflects the early sensory processing of stimulus present to a specific location (Luck et al., 1990; Lin et al., 2019). Previous studies have shown that P1 amplitude enhancement is linked to the attention-related sensory gain control in the early stage of visual processing (Hillyard et al., 1998; Finnigan et al., 2011). The temporal cortex was claimed to be involved in the early stages of feature processing and perceptual discrimination (Lambert and Wootton, 2017). In the present study, P1 amplitude was more positive in the real TMS condition, suggesting the pSTS-TMS facilitates the early stage of feature discrimination, which may result from enhanced sensory gain control through the activation of pSTS.

### N270

N270 amplitude was reduced in the real TMS condition with all mismatch types, while no difference was observed with the match type. This is similar to the previous finding that the pSTS showed greater activation in response to incongruent vs. congruent stimulus pairs and bimodal vs. unimodal conflict (Hocking and Price, 2008). The pSTS plays an important role in conflicts related to face and orientation processing (Eacott et al., 1993), and the results show that pSTS is also involved in color and shape discrimination, as TMS of this brain area facilitated the processing of mismatched stimulus features to a greater extent than that of matched features.

It is well-established that the capacity for processing information is limited. Smaller N270 amplitude reflects reduced neuronal activities and less effort in conflict processing (Wang et al., 2001; Scannella et al., 2016). More attention is required to process conflicting or mismatched information as compared to matched information. The efficient and effective allocation of attentional resources during conflict processing is important for achieving optimal performance (Kramer et al., 1983). In the present study, TMS of the pSTS decreased the amplitude of N270 and shortened RTs, suggesting that less cognitive effort was needed for correct responses with pSTS-TMS. Thus, TMS of the pSTS preferentially enhanced the processing of conflicting over matched information by increasing the availability and efficiency of neurons involved in feature discrimination.

### P3

The P3 component is elicited by cognitive tasks that engage attention after an unexpected stimulus (Polich, 1998). P3 amplitude reflects the mental representations of sensory stimuli at the final stage of information processing. In the present study, there was no difference in P3 amplitude between the sham and real TMS conditions, indicating that the pSTS is not involved in the late stage of visual information processing.

### Time-Varying Network

In the present study, a comparison of the time-varying network changes between the sham and real TMS conditions revealed that TMS of the right pSTS enhanced information flow in the frontal pole, especially with mismatched stimuli.

Previous studies revealed that the frontal pole was associated with cognitive functions such as selective attention (Burgess et al., 2007) and multitasking (Gilbert et al., 2006). The frontal pole cortex is presumed to play a role in tracking and evaluating competing stimuli and is coactivated with the default mode network (Euston et al., 2012). A previous study also showed that patients with right frontal–temporal lobe brain damage have difficulty in performing visual searches due to deficits in feature-based control (Kumada and Hayashi, 2006).

The results of this study suggest that the frontal pole plays a key role in feature discrimination. TMS of the right pSTS activated bilateral frontal poles and enhanced their connectivity with other brain regions, especially the temporo-parietal and occipital regions. This confirms that TMS can modulate neuronal activity beyond the site of stimulation, impacting a distributed network of brain areas (Ferreri et al., 2011). The information flow and formation of a dynamic attention network with the frontal pole as the core were essential for color and shape discrimination, with the pSTS facilitating attentional processing by increasing information flow in the frontal pole.

## Limitations

The present study had some limitations. First, the experimental conditions were relatively limited in terms of target regions (e.g., vertex or motor cortex) and stimulation conditions. Second, as is common in TMS–EEG studies, the TMS pulse caused a high-amplitude artifact in the EEG signals that lasted 200 ms, which was beyond the scope of our analysis. However, we performed ICA and discarded the signal for 60 ms after each pulse to remove these artifacts. Nonetheless, future studies should address these limitations.

## Conclusion

In the present study, we investigated the effects of single-pulse TMS over the right pSTS in the dual-feature delayed matching task. We found that the right pSTS activation improved task performance, especially with mismatched types, and reduced N270 amplitude. The results of the time-varying network analysis revealed that pSTS-TMS altered the dynamic attention network by increasing the connectivity between bilateral frontal poles and the temporo-parieto-occipital regions. These findings provide evidence that the pSTS plays an important role in the feature discrimination aspect of attentional processing.

## Data Availability Statement

The original contributions presented in the study are included in the article/supplementary material, further inquiries can be directed to the corresponding author/s.

## Ethics Statement

The studies involving human participants were reviewed and approved by Ethics Committee of the Xuanwu Hospital, Capital Medical University. The patients/participants provided their written informed consent to participate in this study.

## Author Contributions

YW conceived and designed the study. QZ acquired the data and drafted the manuscript. PS, XW, and HL contributed to data analysis and interpretation. All authors contributed to the article and approved the submitted version.

## Conflict of Interest

The authors declare that the research was conducted in the absence of any commercial or financial relationships that could be construed as a potential conflict of interest.

## References

[B1] Barker A. T. Jalinous R. (1985). Non-invasive magnetic stimulation of human motor cortex. Lancet 1, 1106–1107. 10.1016/S0140-6736(85)92413-4 2860322

[B2] Beauchamp M. S. (2011). “Biological motion and multisensory integration: the role of the superior temporal sulcus,” in The Science of Social Vision, ed R. B. Adams (New York, NY: Oxford University Press), 409–420. 10.1093/acprof:oso/9780195333176.003.0024

[B3] Beauchamp M. S. Yasar N. E. Frye R. E. Ro T. (2008). Touch, sound and vision in human superior temporal sulcus. Neuroimage 41, 1011–1020. 10.1016/j.neuroimage.2008.03.015 18440831 PMC2409200

[B4] Bergmann T. O. Karabanov A. Hartwigsen G. Thielscher A. Siebner H. R. (2016). Combining non-invasive transcranial brain stimulation with neuroimaging and electrophysiology: current approaches and future perspectives. Neuroimage 140, 4–19. 10.1016/j.neuroimage.2016.02.012 26883069

[B5] Bogadhi A. R. Bollimunta A. Leopold D. A. Krauzlis R. J. (2018). Brain regions modulated during covert visual attention in the macaque. Sci Rep. 8:15237. 10.1038/s41598-018-33567-9 30323289 PMC6189039

[B6] Bogadhi A. R. Bollimunta A. Leopold D. A. Krauzlis R. J. (2019). Spatial attention deficits are causally linked to an area in macaque temporal cortex. Curr. Biol. 29, 726–736.e4. 10.1016/j.cub.2019.01.028 30773369 PMC6401289

[B7] Burgess P. W. Dumontheil I. Gilbert S. J. (2007). The gateway hypothesis of rostral prefrontal cortex (area 10) function. Trends Cogn Sci. 11, 290–298. 10.1016/j.tics.2007.05.004 17548231

[B8] Cappa S. F. Sandrini M. Rossini P. M. Sosta K. Miniussi C. (2002). The role of the left frontal lobe in action naming: rTMS evidence. Neurology 59, 720–723. 10.1212/WNL.59.5.720 12221163

[B9] Carter C. S. Macdonald A. M. Botvinick M. Ross L. L. Stenger V. A. Noll D.. (2000). Parsing executive processes: strategic vs. evaluative functions of the anterior cingulate cortex. Proc. Natl. Acad. Sci. U.S.A. 97, 1944–1948. 10.1073/pnas.97.4.1944 10677559 PMC26541

[B10] Delorme A. Makeig S. (2004). EEGLAB: an open source toolbox for analysis of single-trial EEG dynamics including independent component analysis. J. Neurosci. Methods 134, 9–21. 10.1016/j.jneumeth.2003.10.009 15102499

[B11] Eacott M. J. Heywood C. A. Gross C. G. Cowey A. (1993). Visual-discrimination impairments following lesions of the superior temporal sulcus are not specific for facial stimuli. Neuropsychologia 31, 609–619. 10.1016/0028-3932(93)90055-5 8341417

[B12] Euston D. R. Gruber A. J. McNaughton B. L. (2012). The role of medial prefrontal cortex in memory and decision making. Neuron 76, 1057–1070. 10.1016/j.neuron.2012.12.002 23259943 PMC3562704

[B13] Ferreri F. Pasqualetti P. Maatta S. Ponzo D. Ferrarelli F. Tononi G.. (2011). Human brain connectivity during single and paired pulse transcranial magnetic stimulation. Neuroimage 54, 90–102. 10.1016/j.neuroimage.2010.07.056 20682352

[B14] Finnigan S. O'Connell R. G. Cummins T. D. Broughton M. Robertson I. H. (2011). ERP measures indicate both attention and working memory encoding decrements in aging. Psychophysiology 48, 601–611. 10.1111/j.1469-8986.2010.01128.x 21039584

[B15] Gilbert S. J. Spengler S. Simons J. S. Steele J. D. Lawrie S. M. Frith C. D.. (2006). Functional specialization within rostral prefrontal cortex (area 10): a meta-analysis. J. Cogn. Neurosci. 18, 932–948. 10.1162/jocn.2006.18.6.932 16839301

[B16] Hallett M. (2007). Transcranial magnetic stimulation: a primer. Neuron 55, 187–199. 10.1016/j.neuron.2007.06.026 17640522

[B17] Hein G. Knight R. T. (2008). Superior temporal sulcus-it's my area: or is it? J. Cogn. Neurosci. 20, 2125–2136. 10.1162/jocn.2008.20148 18457502

[B18] Hillyard S. A. Vogel E. K. Luck S. J. (1998). Sensory gain control (amplification) as a mechanism of selective attention: electrophysiological and neuroimaging evidence. Philos. Trans. R. Soc. Lond. B Biol. Sci. 353, 1257–1270. 10.1098/rstb.1998.0281 9770220 PMC1692341

[B19] Hocking J. Price C. J. (2008). The role of the posterior superior temporal sulcus in audiovisual processing. Cereb Cortex. 18, 2439–2449. 10.1093/cercor/bhn007 18281303 PMC2536697

[B20] Hyvärinen A. Oja E. (2000). Independent component analysis: algorithms and applications. Neural Netw. 13, 411–430. 10.1016/S0893-6080(00)00026-5 10946390

[B21] Ilmoniemi R. J. Kicic D. (2010). Methodology for combined TMS and EEG. Brain Topogr. 22, 233–248. 10.1007/s10548-009-0123-4 20012350 PMC2800178

[B22] Katsuki F. Constantinidis C. (2014). Bottom-up and top-down attention: different processes and overlapping neural systems. Neuroscientist 20, 509–521. 10.1177/1073858413514136 24362813

[B23] Kramer A. F. Wickens C. D. Donchin E. (1983). An analysis of the processing requirements of a complex perceptual-motor task. Hum. Fact. 25, 597–621. 10.1177/001872088302500601 6671646

[B24] Kumada T. Hayashi M. (2006). Deficits in feature-based control of attention in a patient with a right fronto-temporal lesion. Cogn. Neuropsychol. 23, 401–423. 10.1080/02643290542000085 21049337

[B25] Lambert A. J. Wootton A. (2017). The time-course of activation in the dorsal and ventral visual streams during landmark cueing and perceptual discrimination tasks. Neuropsychologia 103, 1–11. 10.1016/j.neuropsychologia.2017.07.002 28688854

[B26] Lin Y. Cui S. Du J. Li G. He Y. Zhang P.. (2019). N1 and P1 components associate with visuospatial-executive and language functions in normosmic parkinson's disease: an event-related potential study. Front. Aging Neurosci. 11:18. 10.3389/fnagi.2019.00018 30804778 PMC6370661

[B27] Luck S. J. (2014). An Introduction to the Event-related Potential Technique. Cambridge, MA: The MIT Press.

[B28] Luck S. J. Heinze H. J. Mangun G. R. Hillyard S. A. (1990). Visual event-related potentials index focused attention within bilateral stimulus arrays. II. Functional dissociation of P1 and N1 components. Electroencephalogr. Clin. Neurophysiol. 75, 528–542. 10.1016/0013-4694(90)90139-B 1693897

[B29] Majerus S. Peters F. Bouffier M. Cowan N. Phillips C. (2018). The dorsal attention network reflects both encoding load and top-down control during working memory. J. Cogn. Neurosci. 30, 144–159. 10.1162/jocn_a_01195 28984526

[B30] Mao W. Wang Y. (2008). The active inhibition for the processing of visual irrelevant conflict information. Int. J. Psychophysiol. 67, 47–53. 10.1016/j.ijpsycho.2007.10.003 17999937

[B31] Moore T. Zirnsak M. (2017). Neural mechanisms of selective visual attention. Annu. Rev. Psychol. 68, 47–72. 10.1146/annurev-psych-122414-033400 28051934

[B32] Pascual-Leone A. Walsh V. Rothwell J. (2000). Transcranial magnetic stimulation in cognitive neuroscience - virtual lesion, chronometry, and functional connectivity. Curr. Opin. Neurobiol. 10, 232–237. 10.1016/S0959-4388(00)00081-7 10753803

[B33] Pellicciari M. C. Veniero D. Miniussi C. (2017). Characterizing the cortical oscillatory response to tMS Pulse. Front. Cell Neurosci. 11:38. 10.3389/fncel.2017.00038 28289376 PMC5326778

[B34] Polich J. (1998). P300 clinical utility and control of variability. J. Clin. Neurophysiol. 15, 14–33. 10.1097/00004691-199801000-00004 9502510

[B35] Rogasch N. C. Fitzgerald P. B. (2013). Assessing cortical network properties using TMS-EEG. Hum. Brain Mapp. 34, 1652–1669. 10.1002/hbm.22016 22378543 PMC6870446

[B36] Sack A. T. Linden D. E. J. (2003). Combining transcranial magnetic stimulation and functional imaging in cognitive brain research: possibilities and limitations. Brain Res. Rev. 43, 41–56. 10.1016/S0165-0173(03)00191-7 14499461

[B37] Scannella S. Pariente J. De Boissezon X. Castel-Lacanal E. Chauveau N. Causse M.. (2016). N270 sensitivity to conflict strength and working memory: a combined ERP and sLORETA study. Behav. Brain Res. 297, 231–240. 10.1016/j.bbr.2015.10.014 26477377

[B38] Shapiro K. A. Pascual-Leone A. Mottaghy F. M. Gangitano M. Caramazza A. (2001). Grammatical distinctions in the left frontal cortex. J. Cogn. Neurosci. 13, 713–720. 10.1162/08989290152541386 11564316

[B39] Siebner H. R. Bergmann T. O. Bestmann S. Massimini M. Johansen-Berg H. Mochizuki H.. (2009). Consensus paper: combining transcranial stimulation with neuroimaging. Brain Stimul. 2, 58–80. 10.1016/j.brs.2008.11.002 20633405

[B40] Stemmann H. Freiwald W. A. (2019). Evidence for an attentional priority map in inferotemporal cortex. Proc. Natl. Acad. Sci. U.S.A. 116, 23797–23805. 10.1073/pnas.1821866116 31685625 PMC6876153

[B41] Taylor M. J. (2002). Non-spatial attentional effects on P1. Clin. Neurophysiol. 113, 1903–1908. 10.1016/S1388-2457(02)00309-7 12464327

[B42] Wang H. Wang Y. Kong J. Cui L. Tian S. (2001). Enhancement of conflict processing activity in human brain under task relevant condition. Neurosci. Lett. 298, 155–158. 10.1016/S0304-3940(00)01757-2 11165430

[B43] Wang Y. Cui L. Wang H. Tian S. Zhang X. (2004). The sequential processing of visual feature conjunction mismatches in the human brain. Psychophysiology 41, 21–29. 10.1111/j.1469-8986.2003.00134.x 14692997

[B44] Wang Y. Tian S. Wang H. Cui L. Zhang Y. Zhang X. (2003). Event-related potentials evoked by multi-feature conflict under different attentive conditions. Exp. Brain Res. 148, 451–457. 10.1007/s00221-002-1319-y 12582828

[B45] Wang Y. Wang H. Cui L. Tian S. Zhang Y. (2002). The N270 component of the event-related potential reflects supramodal conflict processing in humans. Neurosci. Lett. 332, 25–28. 10.1016/S0304-3940(02)00906-0 12377376

[B46] Yang J. Wang Y. (2002). Event-related potentials elicited by stimulus spatial discrepancy in humans. Neurosci. Lett. 326, 73–76. 10.1016/S0304-3940(02)00204-5 12057831

[B47] Zhang L. Liang Y. Li F. Sun H. Peng W. Du P.. (2017). Time-varying networks of inter-ictal discharge g reveal epileptogenic zone. Front. Comput. Neurosci. 11:77. 10.3389/fncom.2017.00077 28867999 PMC5563307

[B48] Zhang R. Hu Z. Roberson D. Zhang L. Li H. Liu Q. (2013). Neural processes underlying the“same”-“different” judgment of two simultaneously presented objects–an EEG study. PLoS One 8:e81737. 10.1371/journal.pone.0081737 24349122 PMC3861320

[B49] Zhang X. Wang Y. Li S. Wang L. (2003). Event-related potential N270, a negative component to identification of conflicting information following memory retrieval. Clin. Neurophysiol. 114, 2461–2468. 10.1016/S1388-2457(03)00251-7 14652106

